# Quality changes and freezing time prediction during freezing and thawing of ginger

**DOI:** 10.1002/fsn3.314

**Published:** 2015-12-02

**Authors:** Poonam Singha, Kasiviswanathan Muthukumarappan

**Affiliations:** ^1^ Department of Agricultural and Biosystems EngineeringSouth Dakota State UniversityBrookingsSouth Dakota57007

**Keywords:** Essential oil, freezing, ginger, microstructure, quality, simulation

## Abstract

Effects of different freezing rates and four different thawing methods on chemical composition, microstructure, and color of ginger were investigated. Computer simulation for predicting the freezing time of cylindrical ginger for two different freezing methods (slow and fast) was done using ANSYS ^®^ Multiphysics. Different freezing rates (slow and fast) and thawing methods significantly (*P* < 0.05) affected the color and composition of essential oil in ginger. Fresh ginger was found to contain 3.60% gingerol and 18.30% zingerone. A maximum yield of 7.43% gingerol was obtained when slow frozen gingers when thawed by infrared method. Maximum zingerone content of 38.30% was achieved by thawing slow frozen gingers using infrared‐microwave method. Microscopic examination revealed that structural damage was more pronounced in slow frozen gingers than fast frozen gingers. Simulated freezing curves were in good agreement with experimental measurements (*r* = 0.97 for slow freezing and *r* = 0.92 for fast freezing). Slow freezing damaged ginger's cellular structure. Data obtained will be helpful in selecting appropriate thawing method to increase desirable essential oil components in ginger. Computer simulation for predicting freezing time may help in developing proper storage system of ginger.

## Introduction

Ginger (*Zingiber officinale*) is regarded as one of the most important spices grown in the world. It is extensively grown in the tropical and subtropical regions of the world particularly in Bangladesh, India, Taiwan, Jamaica, Africa, Mexico, China, and Japan (Thompson et al. 1973; Baliga et al. 2011; Aziz et al. 2012; Mishra et al. 2013). It is not only used as a spice, but also as a traditional medicine against several diseases. It has been used as a remedy to dyspepsia, nausea, indigestion, colic, and diarrhea (Aziz et al. 2012). The health‐promoting functionality of ginger is often attributed to its rich phytochemistry (Shukla and Singh 2007). The constituents of ginger are numerous and vary depending on the place of origin and form of rhizomes, for example, fresh or dry. The rhizomes contain highly valued aromatic, volatile, and pungent compounds. The nonvolatile components of the ginger are mainly responsible for imparting its pungency, whereas the volatile components are responsible for its aroma (Govindarajan and Connell 1983). The chemical investigations carried out in the past showed that monoterpene hydrocarbons, oxygenated monoterpenes, sesquiterpene hydrocarbons, and nonterpenoid compounds were the main constituents in ginger oils (Onyenekwe and Hashimoto 1999; Kim and Lee 2006; Aziz et al. 2012). Among the many components, *α*‐zingiberene is the most predominant component of ginger oil (Ravindran and Babu 2005). Gingerols are attributed for ginger‐specific pungency and possess substantial antioxidant activity as determined by various antioxidant assays (Butt and Sultan 2011).

Like all fresh fruits and vegetables, ginger is perishable because of its high moisture content. The optimum conditions for storing fresh ginger roots are 13–15°C and 90–95% relative humidity (Enyama 1981; Choi and Kim 2001). For ginger growers it is costly and difficult to maintain the optimum storage conditions of ginger roots and hence, they store them in underground tunnels, where the optimum temperature and humidity control are impossible. This results in spoilage and the ginger roots sprout after a few months (Lee et al. 1994). Ginger roots when gamma irradiated with up to 80 Gy and stored at 25–28°C showed a deterioration in external appearance after 1 month, and shrinkage and discoloration after 2 months (Yusof 1990). Similar results were reported by (Gonzalez et al. 1969), Sirikulvadhana and Prompubesara (1979) and Queirol et al. (2002). Other pretreatments, such as a citric acid treatment (Brown and Lloyd 2007), wax coating (Okwuowulu and Nnodu 1988) and antimicrobial treatment (Subramanyam et al. 1962) exhibited limited effects on extending the storage life of ginger roots, and were not found to be suitable for long‐term storage.

Developing methods for the long‐term storage of harvested ginger roots without a loss in quality is very important to ginger growers as well as to people who process food, and who need a continuous supply of good quality ginger roots throughout the year. It appears that freezing could be a suitable method to preserve the quality of the ginger roots for a long period.

Freezing is an efficient process of preserving the quality of food because in frozen state, water is immobilized as ice and the rates of deterioration are much slower than at higher temperatures. Two major thermal events occur during freezing. At first there is formation of ice crystals (or nucleation) followed by increase in crystal size (crystal growth). The rate of crystal growth is determined by three factors: rate of reaction at the crystal surface, diffusion rate of water to the growing crystal, and rate of heat removal. Crystal size varies inversely with the number of nuclei. Freezing stands on two basic prerequisites to deliver high quality products: (1) rapid freezing rates; and (2) rapid thawing rates (Petzold and Aguilera 2009). Rapid freezing can be accomplished using cryogenic systems employing liquefied gases such as nitrogen or carbon dioxide as refrigerant. Liquid nitrogen has a boiling point of −196°C at atmospheric pressure and the cooling effect is almost instantaneous when sprayed on food stuff. Liquid carbon dioxide when released at atmospheric pressure creates 50% dry ice (solid CO_2_) and 50% vapor, both at −70°C. Dry ice has an extremely high rate of heat removal from food product surface, which can often more than compensate for the higher temperatures compared with liquid nitrogen freezing. The use of CO_2_ freezing depends on the individual application (George 1997).

Frozen storage should markedly enhance storage life. However, freezing process may cause severe changes to tissues, resulting in excessive softening (Delgado and Rubiolo 2005). Complications may also arise due to freezing process itself causing alterations to physicochemical characteristics, biochemical quality, and microbiological safety affected adversely by freeze–thaw cycle (Opoku‐Nkoom 2015). The freezing rate is responsible for tissue damage (Fuchigami et al. 1997) and can result in unacceptable or suboptimal product characteristics after thawing. It is generally accepted that high freezing rates retain the quality better than slow freezing rates (Partmann 1975). However, ultrarapid freezing result in mechanical cracking(Kalichevsky 1995) particularly in large samples with high moisture content and low porosity. (Kim and Hung 1994)

Thawing generally occurs more slowly than freezing. Theoretically, thawing is the inverse process of freezing; they are different not only in phase change direction, cooling and heating process, but also in food freezing time and internal temperature variations (Min 2001). The thawing process is to make the freezing ice melt into water inside the food, and get absorbed by the food to restore the freshness similar to that before frozen. Thawing process is much more complex than the freezing process. During thawing, foods are subject to damage due to chemical and physical changes. Therefore, optimum thawing procedures should be of concern to food technologists ( Fennema et al. 1973; Kalichevsky 1995). Quick thawing of food is desirable to assure food quality of frozen vegetables, bread, pastries and so on. But slow thawing is better for thawing fish and meat (Ji et al. 2014) as it allows to reabsorb much of the moisture from the melting ice crystals so there's less “drip out”. Li and Sun (2002) reviewed on application of novel thawing methods such as microwave thawing, acoustic thawing, and ohmic thawing.

Furthermore, it is essential to predict the temperature and freezing time of foods when designing and evaluating freezing equipment (Mannapperuma and Singh 1989). Freezing time and temperature profile within food can be determined experimentally or predicted approximately by analytical, numerical, or computational simulation methods. Experimental procedures are often too expensive, time consuming and may lack a generalized theoretical description of the process. By comparison, numerical and computational simulation methods based on finite differences and finite element techniques (Mannapperuma and Singh 1989) are more effective in analyzing actual situation. The physical changes of food during phase change have to be understood for proper prediction of its thermal behavior at different temperature conditions. Knowledge of the thermo‐physical properties of food material and surrounding environment such as specific heat and enthalpy, density, thermal conductivity, etc., are required for the prediction of temperature within food (Matuda et al. 2011).

Adjusting the freezing–thawing process variables will help to preserve and retain the quality of the product. No information has been published in the literature on the effects of different thawing methods on the microstructure and chemical composition of ginger. In our study, we employed four different thawing techniques namely room temperature thawing which is a slow thawing process and is also a common household practice to thaw frozen foods, and three quick thawing processes viz. microwave thawing, infrared thawing and infrared‐microwave thawing. Quick thawing maintains the quality of food. During microwave thawing electromagnetic radiation is transmitted through food product which is transferred into heat energy. Infrared thawing uses mechanism of radiation to supply heat which favors high rate of surface heat transfer (Venugopal 2005). The objectives of this study were: (1) to investigate and compare the effects of different freezing and thawing methods on the volatile and nonvolatile contents, the microstructure and appearance of ginger; and (2) to study the temperature profile during freezing and predict freezing time using computer simulation.

## Materials and Methods

### Experimental procedure

#### Freezing

Fresh ginger rhizomes were purchased from local store in Brookings, SD, USA. They were selected to be homogeneous in size and color. The moisture content as determined using AOCS methods (AOAC, 1990) was in the range 85–89% (wb). The gingers were cut into regular cylindrical shape of 40 ± 0.01 mm in length and 8 ± 0.003 mm in diameter. Ginger samples were frozen by two methods: slow freezing in a refrigerator maintained at −18°C and fast freezing by using liquid nitrogen (~203°C). In fast freezing, ginger samples were suspended in closed chamber filled with liquid nitrogen until desired temperature (−18°C) at the geometric center was reached. During freezing (both slow and fast freezing), the temperature profile were recorded at regular intervals, normally every 3–5 sec, using thermocouples inserted at the geometric center of the gingers and connected to a data acquisition system (Personal Daq/56). Freezing rate during slow and fast freezing was 0.24°C·min^−1^ and 1.29°C·sec^−1^ respectively. The core temperature of ginger samples reached −18°C within 28 sec during fast freezing and after 187 min during slow freezing.

#### Thawing

As the final freezing temperature was reached approximately −18°C, the frozen gingers were individually thawed under four different conditions viz. at room temperature (~23°C), in microwave, infrared, and infrared‐microwave condition. Advantinum™ 120 oven (SCA1001KSS02, Louisville, KY) was used for microwave and infrared thawing purposes. During thawing, temperature at the center of the gingers were recorded. Thawing was considered complete when the final core temperature was ~19°C. All treatments and measurements were carried out in triplicate. Thawing time for slow frozen gingers was approximately 87 min when thawed at room temperature (~23°C), 1 min in microwave, 10 min in infrared and 3 min in infrared‐microwave. Thawing time for fast frozen gingers was 27 min at room temperature (~23°C), 56 sec in microwave, 6 min in infrared, and 2 min in infrared‐microwave.

### Physico‐chemical analyses

#### Analysis of essential oil composition

Extraction of essential oil fractions in ginger samples (fresh, frozen, and thawed) was done following a method mentioned elsewhere (Usman et al. 2013). Typically, all ginger samples were dried in oven at 60°C for 24 h and pulverized by using a coffee grinder (SmartGrind, Black & Decker, CH Annex Company, China). Approximately, 0.5 g of each pulverized samples was weighed and placed in a 25 mL volumetric flask with methanol as extracting solvent. The solvents were allowed to percolate the materials which were soaked in it for 48 h before collecting the extract. The extracts were centrifuged at 5000 g for 10 min at room temperature (~23°C) and filtered through a 0.2 *μ* filter prior to analysis.

Analysis was performed using a Gas Chromatography‐Mass Spectrometry (GC‐MS) (Agilent GC–7890A, MSD‐5975C and auto‐sampler‐7693). The capillary columns were 30 m × 0.25 mm × 0.25 mm DB‐5MS (J&W Scientific, Folsom, CA). Ultrapure hydrogen was used as the carrier gas at a flow rate of 1.5 mL·min^−1^ and column head pressure of 52.4 kPa (7.6 psi). The auto‐sampler introduced a 1 *μ*L sample into an injection port with a total inlet flow of 54 mL·min^−1^. The injection port was held at 250°C and contained an Agilent inlet liner of deactivated borosilicate single‐taper with glass wool packing. The purge flow was initiated at 1 min with a flow of 50 mL·min^−1^. The GC oven temperature was initially held at 80°C for 2 min, then elevated at a rate of 9°C·min^−1^ up to 200°C and held for 4 min. The gradient was then increased to 10°C·min^−1^ up to 280°C where it was held constant for 5 min. This gradient resulted in an overall run time of 13 min with ATCA‐(TMS)_3_ eluting at approximately 8.76 min. The GC was interfaced with a mass selective detector with the transfer line held at 265°C. Fragmentation of the sample was as accomplished through electron impact with selected ion monitoring (SIM) mode for monitoring abundant ions of ATCA (m/z 245, 347, and 362) and ATCA‐d2 (m/z 349, and 364) with a dwell time of 100 ms each. The MS conditions were as follows: ion source pressure 2.0 Pa (1.5 × 10 −5 Torr), source temperature 200°C, quadrupole temperature 150°C, electron energy 70 eV, electron emission current 34.6 A, and electron multiplier voltage +400 relative to the autotune setting. The major chemical compositions were identified through a NIST Mass Spectral library.

#### Color measurements

Color measurements of the fresh, frozen, and thawed ginger samples were carried out using Minolta Spectrophotometer (CM‐2500d; Minolta Co. Ltd, Osaka, Japan). The spectrophotometer was first calibrated with a white plate and checked for recalibration between measurements, although no adjustments were necessary. Readings were reported in the L*, a*, b* system. The color values, expressed as L* (whiteness or brightness/darkness), a* (redness/greenness), and b* (yellowness/blueness), for respective samples were determined. Three readings were taken and average values were calculated for each data. Reference color values for the fresh samples ($L_{o}^{*}$ *,* $a_{o}^{*}$ *,* $b_{o}^{*}$) and color values from frozen and thawed samples were employed to determine the changes in each individual color parameters and were calculated as follows:

(1) $$\Delta L^{*}=L^{*}-L_{o}^{*}.$$

(2) $$\Delta a^{*}=a^{*}-a_{o}^{*}.$$

(3) $$\Delta b^{*}=b^{*}-b_{o}^{*}.$$

The total color difference (ΔE*) was determined using the following equation:

(4) $$\Delta E^{*}={\left[\Delta {L^{*}}^{2}+\Delta {a^{*}}^{2}+\Delta {b^{*}}^{2}\right]}^{1/2}.$$

The chroma and hue angle (*H°*, hue angle; red = 0*°*; yellow = 90*°*; green = 180*°*; blue = 270*°*) were also calculated on the basis of the following equations:

(5) $$\text{Chroma}=\sqrt{({a^{*}}^{2}+{b^{*}}^{2}}).$$

(6) $$H^{\circ }=\tan^{-1}\left(b^{*}/a^{*}\right)\;\text{when}\;a^{*}<0\;\text{and}\;b^{*}\geq 0.$$

and

(7) $$H^{\circ }=180+\tan^{-1}\left(b^{*}/a^{*}\right)\;\text{when}\;a^{*}<0.$$

#### Microstructure analysis

Structural observation was carried using a Hitachi‐S3400 N (Tokyo, Japan) scanning electron microscope (SEM) operated at 10 kV. The samples were freeze‐dried to remove water prior to the SEM observation. Freeze‐dried samples approximately 8 mm in diameter and 0.2 mm thickness were mounted on stubs and 10 nm gold was coated using a CrC‐150 sputtering system set to a pressure of 5–10 millitorr. Magnification of 160× was used in all the micrographs in the present study. At least two samples for each treatment showing similar images were used for the results.

### Simulation of freezing process

**Figure 1:**
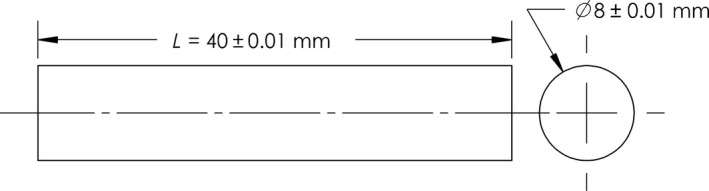
Geometrical dimensions of the ginger: Top view (Left) and Front view (Right).

**Figure 2:**
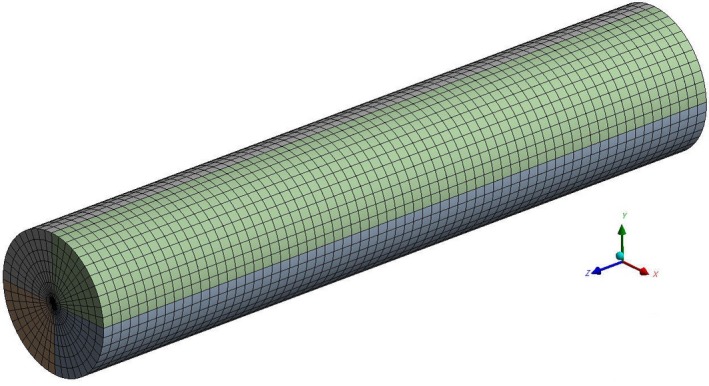
Finite element mesh of ginger model.

The problem considered in this study involves the unsteady one‐dimensional heat transfer in a food (cylindrical in shape) during freezing process. The temperature profile at the core of ginger during freezing (slow and fast) was determined using transient thermal analysis in ANSYS^®^ Multiphysics (ANSYS, Inc., Canonsburg, PA, USA). The geometry of cylindrical ginger was developed using SolidWorks^®^14 (Dassault Systèmes SolidWorks Corporation, Waltham, MA, USA) and its geometrical dimensions are represented in Figure 1. The meshing of the ginger was conducted using ANSYS^®^ Multiphysics. The mesh refinement process was repeated until further mesh refinements have insignificant effects on the results. This process reduces the uncertainties associated with the complexity of heat flow. The generated mesh (Fig. 2) consisted of 19,856 bricks (volume elements). Thermal boundary conditions were applied to the finite element model; (1) free convection from the surface of the ginger and (2) initial temperature of the cylindrical ginger set at 21°C. Following assumptions were made for simulation: (1) heat was transferred radially; (2) the cylindrical ginger was at uniform temperature and was exposed suddenly at time zero to a cooling medium; (3) the cooling medium consisted of air for slow freezing and liquid nitrogen for fast freezing with constant temperature; (4) the food was isotropic; (5) mass transfer between the ginger and the environment was negligible. Inputs for computer simulation were selected to parallel the conditions of the actual experimental freezing trials. A detailed description of input parameters for simulation and thermophysical properties of air and liquid nitrogen are shown in Tables 1 and 2 respectively. The specific heat and thermal conductivity of ginger were measured using KD2 Pro thermal analyzer (Decagon devices, Inc., Pullman, WA, USA).

**Table 1:** Input parameters for simulation of slow and fast freezing of cylindrical ginger

Parameters	Values
Ginger initial temperature (°C)	21
Density of unfrozen ginger, *ρ*_g_ (kg·m^−3^)	1050
Specific heat capacity of unfrozen ginger, Cp_g_ (J·kg^−1^·K^−1^)	3100
Thermal conductivity of air, *k*_air_ (W·m^−1^·K^−1^)	0.8
Air temperature (°C)	−18
Liquid nitrogen temperature (°C)	−203

**Table 2:** Thermophysical properties of air and liquid nitrogen (Anzaldua‐morales et al. 1999; Vasala 2004; Ban et al. 2007; Zachariah 2008; Usman et al. 2013)

	Air (*T* = 255 K)	Liquid nitrogen (*T* = 70 K)
Density, *ρ* (kg·m^−3^)	1.3835	840
Specific heat, Cp (J·kg^−1^·K^−1^)	1003	2024
Viscosity, *μ* (Pa·s)	1.650 × 10^−5^	2.20 × 10^−4^
Thermal conductivity, *k* (W·m^−1^·K^−1^)	0.0228	0.150
Prandtl number, *Pr*	0.715	2.97
Thermal expansion coefficient, *β* (K^−1^)	0.002	0.00504

### Statistical analysis

For data analysis, analysis of variance (ANOVA) was used. Post hoc Tukey's test was used to determine where significant differences (*P* < 0.05) occurred, unless otherwise mentioned. All statistical analysis was performed using SPSS version 16.0 for windows software (SPSS Inc., Chicago, IL).

## Results and Discussion

### Chemical composition of essential oil

**Figure 3:**
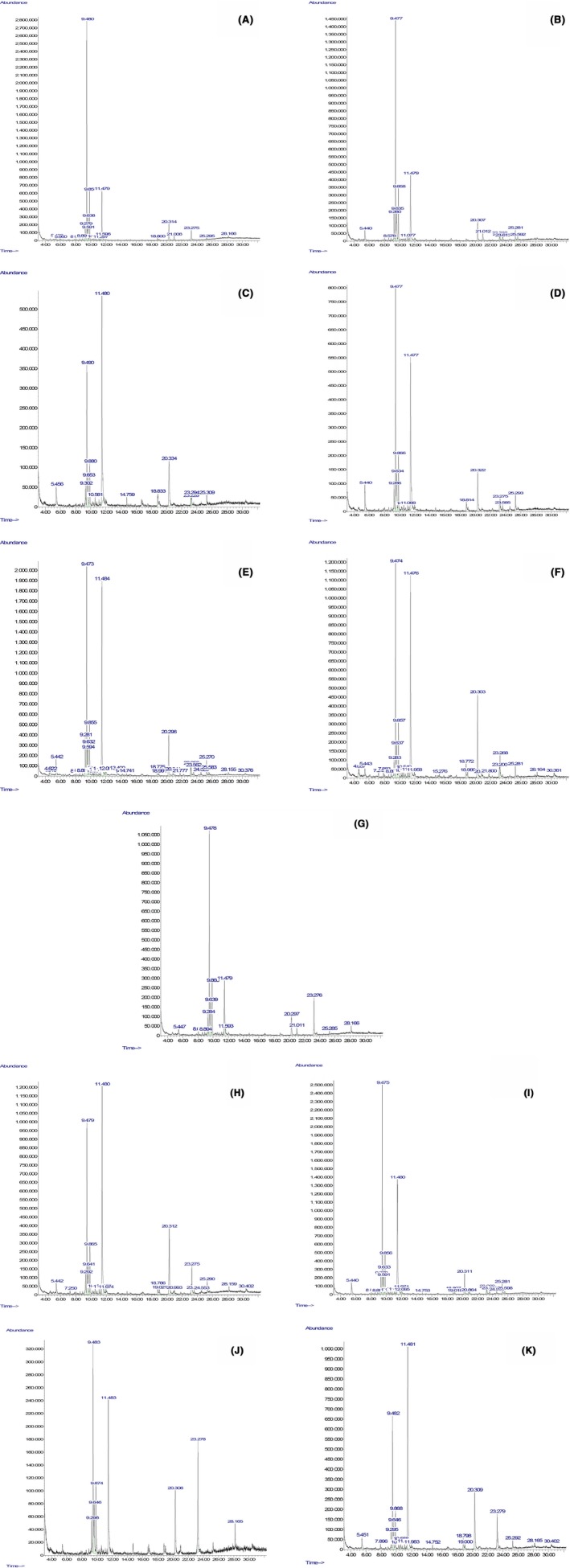
GC‐MS analysis of (A) fresh ginger, (B) fast frozen, (C) fast frozen‐room temperature thawed, (D) fast frozen‐microwave thawed, (E) fast frozen‐infrared thawed, (F) fast frozen‐infrared microwave thawed, (G) slow frozen, (H) slow frozen‐room temperature thawed, (I) slow frozen‐microwave thawed, (J) slow frozen‐infrared thawed, and (K) slow frozen‐infrared microwave thawed.

Volatile and nonvolatile compounds in the essential oils of fresh, frozen, and thawed ginger samples were identified using GC‐MS (Table 3, Fig. 3A). The major volatile and nonvolatile compounds identified were zingiberene (C_15_H_24_), zingerone (C_11_H_14_O_3_), *β*‐sesquiphellandrene (C_15_H_24_), *β*‐bisabolene (C_15_H_24_), curcumene (C_15_H_22_), gingerol (C_17_H_26_O_4_), and farnesene (C_15_H_24_). Fresh ginger essential oil contained 35.5% zingiberene, 18.3% zingerone, 11.3% *β*‐sesquiphellandrene, 6.38% *β*‐bisabolene, 3.6% gingerol, and 1.87% *α*‐farnesene.

**Table 3:** The chemical composition of essential oils of fresh (control), frozen, and thawed gingers (*Zingiber officinale*) analyzed by GC‐MS

Compound	Peak area %										
	FG	SF	FF	SFRT	SFMW	SFIR	SFIR‐MW	FFRT	FFMW	FFIR	FFIR‐MW
Gingerol	3.6^d^	4.2^c^	4.4^c^	2.7^e^	6.6^b^	7.43^a^	6.81^b^	1.5^h^	1.9^f^	1.8^f^	1.7^fg^
Zingerone	18.3^f^	3.6^h^	14^g^	33^b^	27^d^	21.5^e^	38.3^a^	32^bc^	30^cd^	29^cd^	29^c^
Zingiberene	35.5^a^	36^a^	28^c^	19^f^	31^b^	19.7^ef^	17.6^f^	20^ef^	29^bc^	23^d^	22^de^
*β*‐bisabolene	6.38^e^	9.4^a^	8.9^b^	5.3^gh^	6.1^f^	7.13^d^	5.01^i^	5.4^g^	7.9^c^	5.1^hi^	5.3^gh^
*β*‐sesquiphellandrene	11.3^a^	12^a^	11^a^	6.6^cd^	7.8^bc^	8.19^bc^	6.57^cd^	6.7^d^	8.6^b^	6.3^cd^	7.1^bcd^
*α*‐farnesene	1.87^c^	3.3^a^	–	–	2^bc^	–	–	–	–	2.2^b^	–
(E,Z)‐*α*‐farnesene	–	–	–	0.3	–						
(Z,Z)‐*α*‐farnesene	–	–	–	–	0.5						
2,3‐dihydro‐3,5‐dihydroxy‐6‐methyl‐4H‐pyran‐4‐one (DDMP)	0.33^c^	0.2^d^	–	–	–	–	–	–	–	1.2^b^	2.5^a^
Curcumene	4.6^d^	4.9^c^	6.5^a^	3.8^f^	3.8^f^	3.67^f^	3.49^g^	3.1^h^	4.1^e^	5.5^b^	2.8^i^
Copaene	0.45^a^	–	–	–	0.4^a^	–	–	–	–	–	–
Limonene	0.53	–	–	–	–	–	–	–	–	–	–
*α*‐pinene	–	–	3	–	–	–	–	3.2	–	–	–

^a–i^Mean values with different superscript letters are significantly different (*P* < 0.05, Tukey's test).

FG, Fresh ginger; SF, Slow frozen; FF, Fast frozen; SFRT, Slow frozen and room temperature thawed; SFMW, Slow frozen and microwave thawed; SFIR, Slow frozen and infrared thawed; SFIR‐MW, Slow frozen and infrared – microwave thawed; FFRT, Fast frozen and room temperature thawed; FFMW, Fast frozen and microwave thawed; FFIR, Fast frozen and infrared thawed; FFIR‐MW, Fast frozen and infrared – microwave thawed.

The nonvolatile compounds responsible for the pungency in ginger are gingerol, shogaol, and zingerone (Vasala 2004). Shogaol was not detected in fresh, frozen, and thawed ginger essential oils. Gingerol content in essential oil fractions was 4.2% for slow frozen (SF), 4.4% for fast frozen (FF), 6.6% for slow frozen‐microwave thawed (SFMW), 7.43% for slow frozen‐infrared thawed (SFIR), and 6.81% for slow frozen‐infrared microwave thawed (SFIR‐MW). Thawing resulted in increase in gingerone content when compared to fresh and frozen gingers. This increase in zingerone content may be due to transformation of gingerol to zingerone through retro‐aldol reaction at the *β*‐hydroxy ketone group during thawing (Zachariah 2008).

Compounds responsible for the aroma of ginger are *β*‐sesquiphellandrene, zingiberene, *β*‐bisabolene, and farnesene. Zingiberene is the major volatile compound in both fresh and slow frozen (36.27%) gingers. However, zingiberene content decreased in FF (28%), SFMW (31%), FFMW (29%), FFIR (23%), FFIR‐MW (22%), FFRT (20%), SFRT (19%) with maximum decrease found to be in SFIR‐MW (17.6%). *β*‐bisabolene content increased in SF (9.4%), FF (8.9%), SFIR (7.13%), and FFMW (7.9%) compared to that in fresh ginger. *β*‐sesquiphellandrene content in all sample of ginger essential oils decreased except in SF (12%). *α*‐farnesene was not identified in most of the ginger samples. However, *α*‐farnesene content was found to have increased in SF (3.3%), SFMW (2%) and FFIR (2.2%) ginger samples in comparison to that of fresh ginger. Traces of stereoisomers of *α*‐farnesene; (E,Z)‐*α*‐farnesene and (Z,Z)‐*α*‐farnesene were identified in some thawed ginger samples. 2,3‐dihydro‐3,5‐dihydroxy‐6‐methyl‐4H‐pyranone (DDMP; C_6_H_8_O_4_) which has been reported to inhibit colon cancer cell growth (Ban et al. 2007) was found to have increased in the essential oil extract of FFIR‐MW (2.5%). The increase in the volatile and nonvolatile compounds observed in some thawed gingers may be due to more disruption of cell walls and/or influence of heat.

### Color alterations

**Figure 4:**
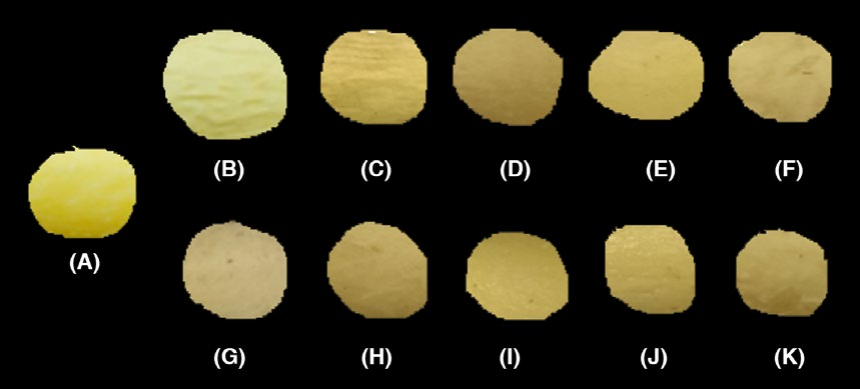
Changes in color of gingers during freezing and thawing: (A) fresh ginger, (B) slow frozen, (C) slow frozen‐room temperature thawed, (D) slow frozen‐microwave thawed, (E) slow frozen‐infra red thawed, (F) Slow frozen‐infrared microwave thawed, (G) fast frozen, (H) Fast frozen‐room temperature thawed, (I) Fast frozen‐microwave thawed, (J) Fast frozen‐infrared thawed, and (K) Fast frozen‐infrared microwave thawed.

**Figure 5:**
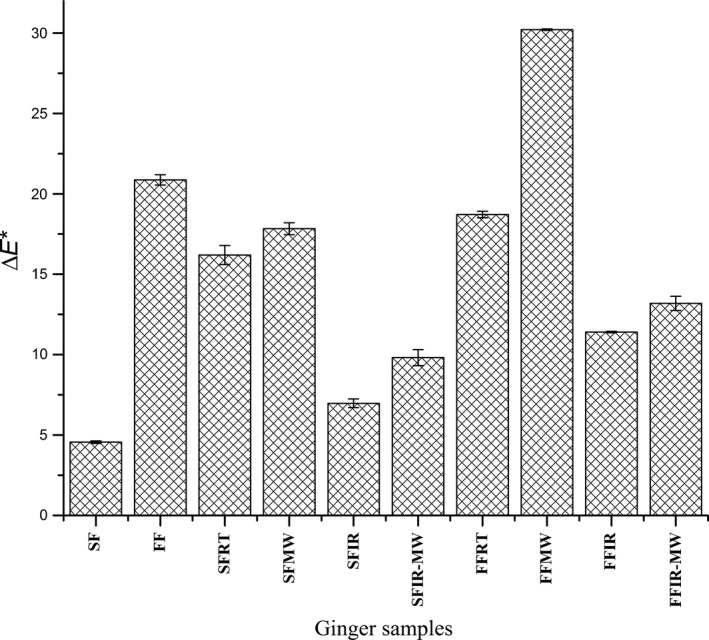
Effect of freezing and thawing on total color difference (ΔE*). SF, Slow frozen; FF, Fast frozen; SFRT, Slow frozen and room temperature thawed; SFMW, Slow frozen and microwave thawed; SFIR, Slow frozen and infrared thawed; SFIR‐MW, Slow frozen and infrared – microwave thawed; FFRT, Fast frozen and room temperature thawed; FFMW, Fast frozen and microwave thawed; FFIR, Fast frozen and infrared thawed; FFIR‐MW, Fast frozen and infrared – microwave thawed.

Color properties of fresh, frozen, and thawed gingers were determined by using CIE (Commission international de l' éclairage) L*a*b* measurements as shown in Table 4. Color is an important attribute and undergoes significant changes during freezing and thawing (Fig. 4). The L* value (lightness) of fresh ginger was 77.08 which decreased (*P* < 0.05) when gingers were frozen and thawed. The decrease in L* value was more pronounced in all fast frozen and thawed gingers and for SFRT and SFMW. The a* value, which is a measure of redness and greenness, was found to decrease (*P* < 0.05) more during slow freezing (−3.11) than during fast freezing (−1.94) when compared to fresh ginger (−1.54). After thawing at room temperature (~23°C), slow frozen gingers showed more yellowness (−1.32) than fresh ginger. However, all other thawing methods significantly decreased (*P* < 0.05) the a* values of both slow and fast frozen gingers. Except for SFRT, all samples showed lower a* value (*P* < 0.05) suggesting more greenness. All frozen and thawed samples had lower b* (*P* < 0.05) value compared to that of fresh ginger (40.45) suggesting loss in yellow color. Hue angle values increased (*P* < 0.05) for all ginger samples except for FF and SFRT. Figure 5 shows the ΔE* value, which was calculated based on the L*, a*, and b* values of ginger according to different freezing and thawing methods. The total color difference (ΔE*) was highest in fast frozen‐microwave thaw (FFMW) and lowest in SF. The mean ΔE* value of SF was significantly lower (*P* < 0.05) than that of other frozen and thawed samples, indicating that the surface color of the SF ginger was closer to that of fresh ginger. The color difference may be explained by the fact that chroma values of all the frozen and thawed gingers was lower than fresh ginger. The chroma value indicates the saturation or color purity and is effected by a* and b* values.

**Table 4:** Color values of fresh (control), frozen, and thawed ginger (*Zingiber officinale*) samples

Parameter	Ginger samples										
	FG	SF	FF	SFRT	SFMW	SFIR	SFIR‐MW	FFRT	FFMW	FFIR	FFIR‐MW
L* (Lightness)	77.08^a^ (0.05)	74.33^b^ (0.07)	58.42^h^ (0.30)	62.08^f^ (0.56)	61.38^fg^ (0.50)	71.51^c^ (0.30)	68.68^d^ (0.64)	59.29^h^ (0.16)	60.42^g^ (0.10)	66.12^e^ (0.13)	66.20^e^ (0.54)
a* (Redness)	−1.54^b^ (0.02)	−3.11^f^ (0.02)	−1.94^c^ (0.01)	−1.32^a^ (0.01)	−3.69^g^ (0.02)	−2.56^e^ (0.03)	−2.54^e^ (0.02)	−3.95^i^ (0.03)	−2.06^d^ (0.02)	−3.06^f^ (0.02)	−3.84^h^ (0.03)
b* (Yellowness)	40.45^a^ (0.17)	37.18^bc^ (0.06)	31.11^h^ (0.11)	34.37^e^ (0.34)	32.29^g^ (0.16)	36.69^c^ (0.14)	35.48^d^ (0.33)	35.18^d^ (0.23)	35.65^d^ (0.29)	37.72^b^ (0.37)	33.38^f^ (0.15)
Chroma	40.48^a^ (0.17)	37.31^bc^ (0.06)	31.20^h^ (0.11)	34.39^e^ (0.34)	32.50^g^ (0.16)	36.78^c^ (0.14)	35.57^d^ (0.33)	35.39^d^ (0.24)	35.71^d^ (0.29)	37.76^b^ (0.48)	33.59^f^ (0.15)
*H* ^*o*^ (Hue angle)	179.55^de^ (0.44)	181.41^b^ (0.28)	176.95^f^ (0.19)	179.10^e^ (0.60)	181.23^b^ (0.02)	180.17^cd^ (0.09)	179.84^de^ (0.11)	181.75^b^ (0.09)	180.02^de^ (0.19)	183.49^a^ (0.95)	181.14^bc^ (0.22)

^a–i^Mean values with different superscript letters are significantly different (*P* < 0.05, Tukey's test). Parentheses indicate ± standard deviation (*n* = 3).

FG, Fresh ginger; SF, Slow frozen; FF, Fast frozen; SFRT, Slow frozen and room temperature thawed; SFMW, Slow frozen and microwave thawed; SFIR, Slow frozen and infrared thawed; SFIR‐MW, Slow frozen and infrared – microwave thawed; FFRT, Fast frozen and room temperature thawed; FFMW, Fast frozen and microwave thawed; FFIR, Fast frozen and infrared thawed; FFIR‐MW, Fast frozen and infrared – microwave thawed.

### Microstructure analysis

**Figure 6:**
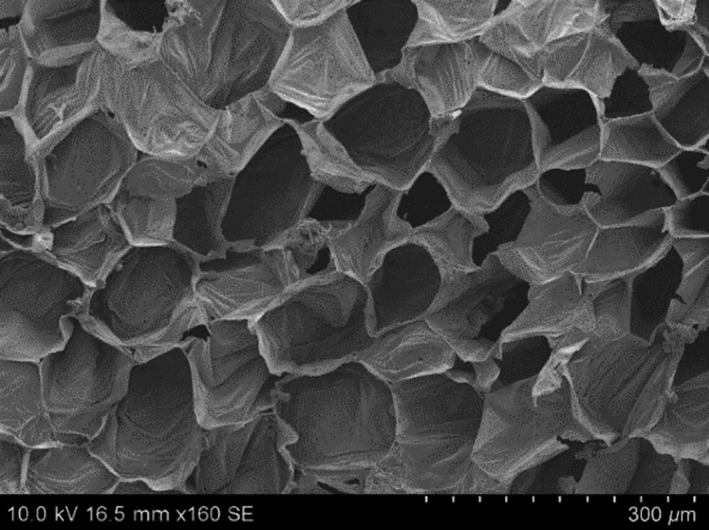
Electron micrograph showing cellular structure of fresh ginger rhizome. Scale bar = 300 *μ*m.

**Figure 7:**
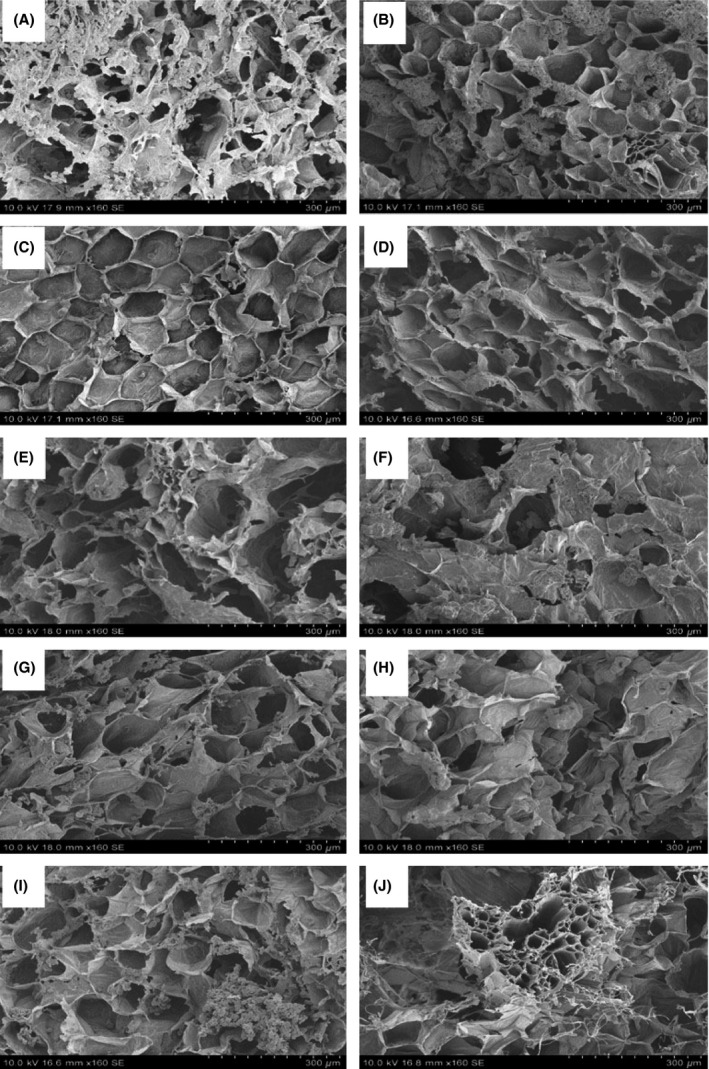
Electron micrograph of (A) slow frozen, (B) fast frozen, (C) slow frozen‐room temperature thawed, (D) slow frozen‐microwave thawed, (E) slow frozen‐infra red thawed, (F) slow frozen‐infrared microwave thawed, (G) fast frozen‐room temperature thawed, (H) fast frozen‐microwave thawed, (I) fast frozen‐infrared thawed, and (J) fast frozen‐infrared microwave thawed gingers. Scale bar = 300 *μ*m.

To gain insight into the effects of freezing and thawing on the structure of ginger, scanning electron microscopic (SEM) images were obtained to provide visual evidence of the changes in structure. Figure 6 shows microscopic image of fresh sample of ginger rhizome, which did not receive any other treatment other than preparation for SEM. The impacts of freezing on quality of food are directly related with the growth of ice crystals which can break cellular walls (Anzaldua‐morales et al. 1999). Ginger rhizome typically contains 85–89% moisture (wb). When ginger was subjected to slow freezing large ice crystals were formed which disrupted the cells. Figure 7A shows the structural damage caused due to formation of large ice crystal during slow freezing. Contrary to this, fast or rapid freezing leads to formation of smaller ice crystals and hence causes minimum damage to cellular structure (Fig. 7B). Rapid freezing is appropriate to retain the tissue structure. This is in agreement with Delgado and Rubiolo (2005).

Thawing also plays an important role in regulating the cellular structure of food. In our study, we investigated the effects of different thawing process on the microstructure of ginger. When gingers are thawed, the cells try to resume or regain their original shape. However, this greatly depends on the thawing method. During thawing reabsorption of water and other soluble substances by the cells takes place. From the SEM images (Fig. 7C–J), it can be said that after reabsorption, water and other products are better distributed within the cellular compartments of the fast frozen gingers than those of slow frozen ones. The reason behind such phenomenon could be due to less cell damage occurring during fast freezing than while slow freezing.

### Freezing time prediction

**Figure 8:**
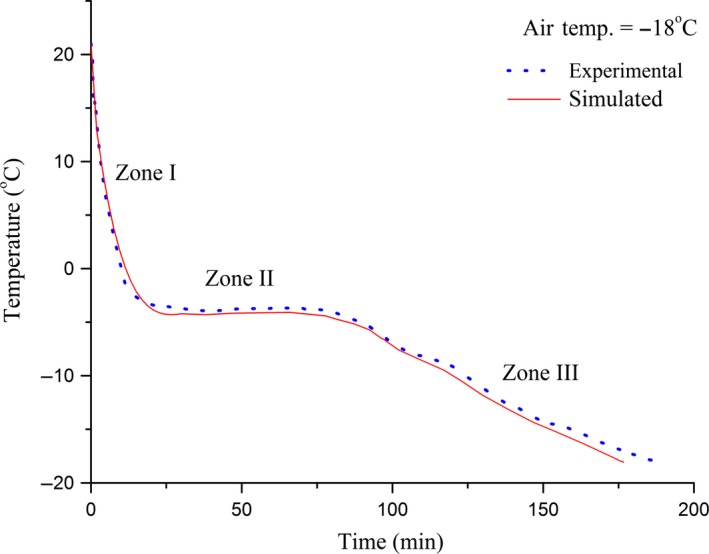
Comparison of simulated (solid line) and experimental (dotted line) results for slow freezing of ginger (*r* = 0.97).

**Figure 9:**
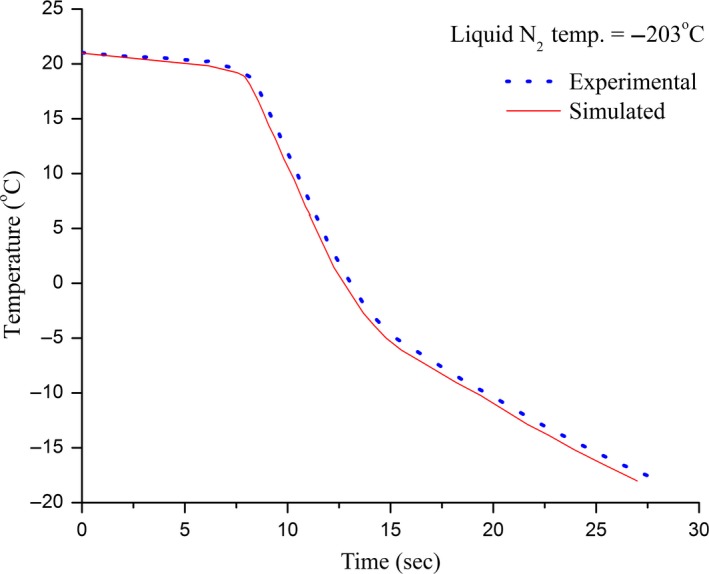
Comparison of simulated (solid line) and experimental (dotted line) results for fast freezing of ginger (*r* = 0.92).

Verification of the simulated results for the freezing time prediction was achieved by comparing with the experimental data obtained. Good correlation (*r* = 0.97 for slow freezing and *r* = 0.92 for fast freezing) was found between experimental measurements and the simulated results. The freezing curves for slow and fast freezing of ginger are shown in Figures 8 and 9, plotted over the simulated temperature history (solid line). From the figures it can be seen that the simulated curves closely predicts the actual freezing curves. Simulated curves slightly deviate from experimental data but are positioned quite closely to the observation for the practical‐need accuracy. In case of slow freezing, at temperatures <−5°C the prediction method computed freezing rate ~1.06× faster than those determined experimentally. This amounts to freezing time difference of 9 min. On the other hand for fast freezing, at temperatures <−5°C the prediction method computed freezing rate ~1.39× faster than those determined experimentally. This amounts to freezing time difference of 2 sec. Variation in temperature measurements was attributed to uncertainty in the thermocouple position and limited control over freezing conditions. Precautions were taken during measurements to minimize possible influence of these factors. Taking all the uncertainties with the experimental results in to consideration the prediction method adequately simulates the freezing process.

## Conclusions

All freezing and thawing methods to which the ginger samples were subjected caused changes in color and alteration in internal structure. Slow frozen gingers suffered more damage due to large ice crystal formation than fast frozen gingers. These structural changes affected the chemical compounds identified in the essential oils of frozen and thawed gingers. Rapid freezing is desirable in restoring the structure of food materials but slow freezing may contribute in increasing the chemical compounds of ginger essential oil. Simulated freezing curves closely modelled corresponding experimental freezing curves. Predicted freezing time for ginger during slow and fast freezing differed from experimental result by 9 min and 2 sec respectively. This study shows how different freezing and thawing methods affects the structure of ginger thereby playing a decisive role in enhancing some aromatic and pharmacologically important components of ginger. Also, computer simulation for predicting the temperature and freezing time of ginger will be an important tool in designing and evaluating freezing equipment.

## Conflict of Interest

None declared.
